# Physical activity patterns across time-segmented youth sport flag football practice

**DOI:** 10.1186/s12889-018-5108-3

**Published:** 2018-02-08

**Authors:** Chelsey R. Schlechter, Justin M. Guagliano, Richard R. Rosenkranz, George A. Milliken, David A. Dzewaltowski

**Affiliations:** 1 Department of Kinesiology, Kansas State University, Natatorium 1a, Manhattan, KS 66506 USA; 2 College of Public Health, University of Nebraska Medical Center, 984365 Nebraska Medical Center, Omaha, NE 68198-4365 USA; 3 Department of Food, Nutrition, Dietetics and Health, Kansas State University, 213 Justin Hall, Manhattan, KS 66506 USA; 4 Department of Statistics, Kansas State University, 101 Dickens Hall, Manhattan, KS 66506 USA; 5 Buffett Early Childhood Institute, University of Nebraska, 2111 S. 67th Street, Suite 350, Omaha, NE 68106 USA

**Keywords:** Moderate-to-vigorous physical activity, Organized sport, Youth, Lesson context, Direct observation, Video observation

## Abstract

**Background:**

Youth sport (YS) reaches a large number of children world-wide and contributes substantially to children’s daily physical activity (PA), yet less than half of YS time has been shown to be spent in moderate-to-vigorous physical activity (MVPA). Physical activity during practice is likely to vary depending on practice structure that changes across YS time, therefore the purpose of this study was 1) to describe the type and frequency of segments of time, defined by contextual characteristics of practice structure, during YS practices and 2) determine the influence of these segments on PA.

**Methods:**

Research assistants video-recorded the full duration of 28 practices from 14 boys’ flag football teams (2 practices/team) while children concurrently (*N* = 111, aged 5–11 years, mean 7.9 ± 1.2 years) wore ActiGraph GT1M accelerometers to measure PA. Observers divided videos of each practice into continuous context time segments (*N* = 204; mean-segments-per-practice = 7.3, *SD* = 2.5) using start/stop points defined by change in context characteristics, and assigned a value for task (e.g., management, gameplay, etc.), member arrangement (e.g., small group, whole group, etc.), and setting demand (i.e., fosters participation, fosters exclusion). Segments were then paired with accelerometer data. Data were analyzed using a multilevel model with segment as unit of analysis.

**Results:**

Whole practices averaged 34 ± 2.4% of time spent in MVPA. Free-play (51.5 ± 5.5%), gameplay (53.6 ± 3.7%), and warm-up (53.9 ± 3.6%) segments had greater percentage of time (%time) in MVPA compared to fitness (36.8 ± 4.4%) segments (*p* ≤ .01). Greater %time was spent in MVPA during free-play segments compared to scrimmage (30.2 ± 4.6%), strategy (30.6 ± 3.2%), and sport-skill (31.6 ± 3.1%) segments (*p* ≤ .01), and in segments that fostered participation (36.1 ± 2.7%) than segments that fostered exclusion (29.1 ± 3.0%; *p* ≤ .01). Significantly greater %time was spent in low-energy stationary behavior in fitness (15.7 ± 3.4%) than gameplay (4.0 ± 2.9%) segments (*p* ≤ .01), and in sport-skill (17.6 ± 2.2%) than free-play (8.2 ± 4.2%), gameplay, and warm-up (10.6 ± 2.6%) segments (*p* < .05).

**Conclusions:**

The %time spent in low-energy stationary behavior and in MVPA differed by characteristics of task and setting demand of the segment. Restructuring the routine of YS practice to include segments conducive to MVPA could increase %time spent in MVPA during practice. As YS reaches a large number of children worldwide, increasing PA during YS has the potential to create a public health impact.

## Background

Current public health physical activity guidelines recommend that children accrue 60 min of moderate-to-vigorous physical activity (MVPA) per day to achieve overall health benefits, including decreased cardiovascular and metabolic disease risk factors, increased bone density, muscular and cardiovascular fitness [1]. Despite these health benefits, surveillance estimates have indicated that only 42% of children and 8% of adolescents in the United States are meeting physical activity guidelines [2].

Of the 60 min of MVPA that children are suggested to accumulate daily, 30 min are recommended to come from time spent at school, and the remaining 30 min from out-of-school time [3]. One out-of-school setting that provides an opportunity to accumulate MVPA is youth sport [4, 5]. Participation in youth sport has been shown to provide a myriad of psychological, social, and physical benefits for children, including increased health-related quality of life [6], social integration [7], and confidence [7]. In addition, children have been shown to accumulate more physical activity (PA) on youth sport days compared to non-sport days [8]. Despite providing an opportunity to accrue MVPA, much of youth sport time is spent sedentary or in light activity [8–12]. Many children worldwide participate in youth sport [13], therefore targeting YS as a setting to increase PA has the potential to reach a large number of children.

To identify periods of time when children are active and inactive, researchers have characterized the pattern of children’s PA by segmenting across time and by contextual characteristics [14–19]. Within the day, children’s PA has been segmented into morning and afternoon [16], before, during, and after-school [14, 19], indoor and outdoor [15–17], specific class periods such as recess and PE [8, 18], and hour by hour [19]. Despite the ability of accelerometers to provide a detailed time-stamped assessment of the pattern of PA during shorter time frames such as the duration of a youth sport practice, to date studies reporting PA and context during youth sport have only reported mean activity and total percentage of time spent in various contextual conditions for the entire practice [9, 10]. Though reporting average activity and context across an entire practice provides valuable information as to how practice time is spent, averaging PA across an entire practice does not allow for examining the heterogeneous peaks and valleys of activity that occur during a youth sport practice, and thus restricts researchers’ ability to determine the processes that could be causing the variability in practice PA. Each youth sport practice can be considered a dynamic social system with multiple ecological processes that influence children’s PA [20]. Examination of the continuous pattern of PA in synchrony with contextual characteristics during a youth sport practice is important to determine periods of time spent active or inactive during practice and in turn identify the ecological processes that are driving PA.

Researchers in youth activity settings have identified several ecological processes as potential drivers of PA (or inactivity), including task (e.g., management, free-play, gameplay) [21], the setting member arrangement (e.g., whole group, small group) [16], and the demand of the setting (e.g., fosters participation, fosters exclusion) [22, 23]. In physical education, management and knowledge delivery contexts were found to have negative correlations with boys’ MVPA [21]. In contrast, the authors found that gameplay and free-play both demonstrated a positive correlation with boys’ MVPA [21]. In preschool, children were found to have a higher percentage of time spent in total physical activity while arranged in small group, compared to whole group [16]. In summer camps, elimination games (i.e., games that foster exclusion) have been shown to have lower amounts of MVPA than non-elimination games (i.e., games that foster participation) [23].

The purpose of this study was to describe the type and frequency of segments of time, defined by contextual characteristics, during youth sport (YS) practice, and determine the influence of these segments on physical activity (PA). We hypothesized that (1) practices would have heterogeneous time segments defined by contextual characteristics, (2) member arrangement and setting demand of the time segment would influence MVPA, and (3) time segments that demanded participation (i.e., optimal demand) would result in greater MVPA compared to segments that fostered exclusion (i.e., disadvantaged demand).

## Methods

The protocol for this cross-sectional study (#7289) was approved by the Institutional Review Board of the study authors’ university.

### Setting

Teams were recruited from a convenience sample of youth recreational flag football (i.e., a non-tackle variation of North American football) program run by the local Parks and Recreation of a Midwestern U.S. city (population > 50,000 people). The program was divided into 3 leagues based on grade; all 24 teams in the 1st/2nd and 3rd/4th grade leagues were eligible to participate in the study. Each team was coached by a volunteer, and each coach determined the day, time, location, and duration of practice for the team. Teams practiced 1–2 times/week, and played 1 game/week. The season ran for 8 weeks, from the last week of August until the last week of October.

### Participants

**Fig. 1:**
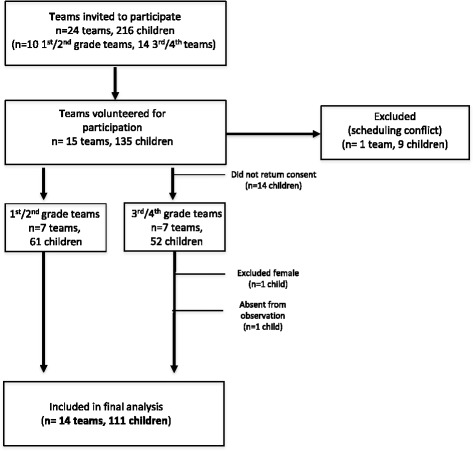
Consent flow diagram

After coaches consented to participate in the study, all players on their team were invited to participate (Fig. 1). A total of 126 boys were eligible for participation, of which 112 provided parental informed consent (91%). Only children with parental consent were included in the study. Analysis included 111, 5–11 year-old boys (mean age ± standard deviation = 7.9 ± 1.2 years). Participant characteristics have been presented elsewhere [12].

### Outcome measures

#### Physical activity

Physical activity was objectively measured using ActiGraph GT1M accelerometers (ActiGraph; Pensacola, FL). ActiGraph accelerometers are the most widely used accelerometers [24] and have been shown to provide a valid and reliable measure of physical activity in youth [25]. To capture the sporadic nature of youth during flag football, accelerometers were initialized to record in 15 s epochs [26] and cut-points by Evenson et al. [27] were applied to determine time spent sedentary (≤100 counts per minute; CPM), light (LPA; 101–2295 CPM), moderate (MPA; 2296–4011 CPM), and vigorous physical activity (VPA; ≥4012 CPM). Evenson cut-points are currently considered the most accurate estimation of PA for this age group [25]. Due to current calls to reach consensus on the definition of the term ‘sedentary’ we will hereafter refer to activity at or below 100 CPM as low-energy stationary behavior rather than sedentary [28].

#### Video observation

Video was recorded using the video capability of Apple™ iPod Touch 5th Generation (California, USA) and a wide-angle lens. Two cameras recorded each practice, a belt-worn camera worn by the head coach and a camera fixed on top of a tripod and positioned to view the whole field.

#### Contextual variables

The coding scheme, definitions, and examples for each contextual variable are presented in Table 1 (See [22]). Each time segment was assigned a value for the task goal of the segment (task), the arrangement of members within the segment (member arrangement), and whether the segment fostered participation or fostered exclusion (setting demand). The categories for each contextual characteristic were determined by a review of the literature [22] and developed according to existing observation systems used for physical activity [9, 29] and education settings [30, 31]. After a preliminary coding system was developed, a subsample of videos were coded by two research assistants for an inter-rater reliability assessment. Then, the research team met to modify existing codes and add any codes that were deemed relevant and necessary to the system.

**Table 1:** Coding scheme, definitions, and examples for each contextual variable

Code	Definition	Example
Task	The purpose of the time segment.	
Warm-up	Time devoted to a routine execution of physical activity with a purpose to prepare the individual for engaging in further activity, but not designed to alter the skill or fitness of the individual on a long-term basis. Usually occurs in the beginning of practice [29]	At the beginning of practice the coach has kids do a serious of dynamic warm-ups and stretches as a group (high knees, lunges, butt kicks, etc.)
Free play	Time during which adult influence of task choice is not intended [29].	The coach has footballs for the kids to play with at the beginning of practice but does not tell the kids what activities to do or not to do.
Fitness	Time where major purpose is to alter the physical state in terms of cardiovascular endurance, strength or flexibility [29, 29].	Running sprints
Sport Skill	Adult-led activity time devoted to practice of skills with the primary goal of skill development [9, 29, 31].	Passing drills, flag grabbing drills
Game play	Adult-led time devoted to playground games where skills are not directly applicable to a competitive sport game and there is little to no adult instruction or feedback [9, 29, 31].	Tag, sharks and minnows
Scrimmage	Adult-led activity time devoted to the refinement and extension of skills in a sport game where two opposing teams are created within a team. Minimal interference from the coach [9, 29, 31].	Within a team, the kids are playing a mock football game
Strategy	Time devoted to transmitting information related to rules and strategy of the sport [29, 31].	Putting in or practicing an offensive play, defensive system, etc.
Management	Time allocated to managerial and organization activities, time devoted to team business that is unrelated to instructional activity [29, 31].	Time out, opening huddle, closing huddle
Self-care	Time devoted to washing, using the rest room, or drinking water.	Water break
Member Arrangement	The arrangement of the setting members within an segment.	
Solitary	Child is doing activity alone [9, 29, 31].	During a dribbling drill, the child is practice by him or herself.
One v One	Child is doing activity with only one additional participant [9].	During a blocking drill, each child has a partner and they take turn blocking.
Small group	Child is performing an activity with greater than one other child, but less than the full team [9].	During a receiving drill, the full team is split into two groups. Each group has their own drill to complete, and the groups are not working together.
Whole group	All children are participating in an activity [9, 29, 31].	All kids go to water break at the same time.
Setting Demand	Population distribution that influences the system	
Optimal	Time period when there are an equal number of opportunities to participate as children to participate (i.e., fosters participation) [20].	During tag all 7 kids are playing at the same time, during warm-up all the kids are on the line at the same time
Disadvantaged	Time period when there are a fewer number of opportunities to participate than children available to participate (i.e., fosters exclusion) [20].	During tag, if you get tagged you have to sit on the sideline until all of the children are out. During a passing drill, only 1 child is receiving the pass at a time, the rest are waiting in line behind him.

### Procedures

For each of the participating teams (*N* = 14) a research assistant attended a practice to introduce the project to parents, familiarize children with the accelerometers, and collect parent and coach consent and survey information. Only children with parental consent were included in the study.

During September and October, one or more research assistants attended two practices per team, allowing at least 14 days between the first and second practice. Upon arrival to the practice, a research assistant set up a tripod and gave the wearable camera to the head coach. Research assistants placed accelerometers on the right hip of each consenting child as he arrived to practice, and removed it upon practice completion. Practice beginning and end times, child’s accelerometer on-and-off times, and video start and stop times were recorded using a universally synchronized clock.

#### Video coding

Videos were uploaded to a video analysis software (NOLDUS, OBSERVER XT 11.5) to code for contextual variables. Each video was divided into naturally occurring time segments, defined by a change in task, member arrangement, or setting demand, then each segment was coded for each of the three variables (i.e., task, member arrangement, and setting demand). Two research assistants who had completed training and demonstrated reliability (≥80% agreement to pre-coded, gold standard video) were randomly assigned one practice per team, with a subsample of videos coded by both research assistants. To ensure inter-rater reliability of the coding scheme, the first four videos (two practices for two teams) were coded by both research assistants and percentage of agreement was calculated. After completion of coding of half of the videos, another four videos (two practices for two teams) were coded by both research assistants, and agreement was again checked to ensure inter-rater reliability remained high. Eight total practices from 4 teams (two practices per team) with a total of 68 segments were coded by both research assistants. Percentage of agreement for start and stop time of segments was 85.3%. Total percentage of agreement for all contextual variables was 91.8%.

#### Data reduction

Using a SAS macro developed by the authors, Evenson cut points [27] were applied to physical activity counts to determine physical activity intensity. After video coding, 15-s accelerometer epoch data were matched with the segment start and stop times derived from video observation and were merged with contextual characteristics from video observation. As a result, each segment was assigned a value for physical activity (derived from team accelerometer data), and a category of the contextual variables of task, member arrangement, and member demand.

### Statistical analysis

All statistical analyses were conducted in SAS (Version 9.4; Cary, NC, USA). Mean and standard deviation were calculated for descriptive characteristics of participants and time segments. A strip plot multilevel model with the interaction of team crossed with day created the time segment as the unit of analysis [32]. This model was used to examine the influence of task, member arrangement, and setting demand on physical activity levels (i.e., low-energy stationary behavior, MVPA, VPA) during practice using SAS PROC MIXED [32]. Team, subject, day, and day-by-team were used as random effects, and significance was set at α = 0.05.

## Results

Mean practice duration was 61.5 (*SD* = 8.6) minutes. Across all teams, 19.9 min (95% CI = 17.6, 22.3) of each practice was spent in MVPA. Approximately 13% (95% CI = 10.8, 15.2) of practice time was spent in low-energy stationary behavior, 34% (95% CI = 31.1, 36.9) of practice time in MVPA, and 12% (95% CI = 10.4, 13.6) of practice time in VPA.

### Segment characteristics

Segment characteristics are presented in Table 2. Across all teams 204 time segments were identified. An average of 7 segments (mean = 7.3, *SD* = 2.5) occurred per practice.

**Table 2:** Segment characteristics

Segment	Frequency		Mean segment length in minutes	Teams including segment type in at least one practice
(n = 204)	% (n)	Number per practice	Mean ± SD (range)	% (n)
Task				
Warm-up	7.84 (16)	0.57	3.39 ± 1.74 (1.50–8.00)	71.43 (10)
Fitness	3.92 (8)	0.33	1.19 ± 0.64 (0.50–2.25)	28.57 (4)
Free-play	3.43 (7)	0.29	5.32 ± 3.21 (1.50–10.75)	35.71 (5)
Game-play	4.41 (9)	0.32	8.14 ± 3.56 (1.25–14.75)	50.00 (7)
Management	18.14 (37)	1.32	1.78 ±1.52 (0.25–9.25)	92.86 (13)
Scrimmage	2.94 (6)	0.21	21.25 ± 15.01 (5.00–47.50)	42.86 (6)
Self-care	16.18 (33)	1.18	1.21 ± 0.50 (0.50–2.25)	92.86 (13)
Sport-skill	24.14 (49)	1.75	9.83 ± 5.87 (2.5–26.75)	100 (14)
Strategy	19.12 (39)	1.39	16.97 ±7.63 (1.50–33.00)	100 (14)
Member Arrangement				
One v One	2.45 (5)	0.18	6.05 ± 3.05 (2.50–9.50)	35.71 (5)
Small group	4.41 (9)	0.32	18.64 ± 7.82 (9.00–33.00)	42.86 (6)
Whole group	93.14 (190)	6.79	7.72 ± 7.85 (0.25–47.50)	100 (14)
Setting Demand				
Disadvantaged	20.12 (34)	1.21	9.63 ± 5.36 (2.00–25.75)	85.71 (12)
Optimal	67.16 (137)	4.82	8.64 ± 8.89 (0.25–47.50)	100 (14)

*SD standard deviation*

### Physical activity by contextual variables

Least-squared means estimates and associations for percentage of time spent in each physical activity intensity for segment types are presented in Table 3.

**Table 3:** Physical activity intensity by segment type

Percentage of time, adjusted mean (95% CI)						
	Low-energy stationary behavior	Differences^a^ (*p* < .05)	VPA	Differences^a^ (*p* < .05)	MVPA	Differences^a^ (*p* < .05)
Task						
a. Warm-up	10.63 (4.79–16.06)	d, e, h	23.40 (19.68–27.12)	e, f, g, h	53.92 (46.84–60.96)	b, e, f, g, h, i
b. Fitness	15.73 (8.84–22.56)	d	20.08 (15.00–25.20)	e, f, g, h, i	36.75 (27.73–45.82)	a, c, d, e
c. Free-play	8.16 (0.00–16.43)	e, h	17.97 (11.53–24.47)	e, h, i,	51.51 (40.72–62.28)	b, e, f, g, h, i
d. Game-play	4.03 (0.00–9.88)	a, b, e, g, h, i,	23.84 (19.49–28.11)	e, f, h, i,	53.56 (46.35–60.85)	b, e, f, g, h, i
e. Management	21.86 (17.59–26.21)	a, c, d, f, g, h, i	10.01 (7.16–13.04)	a, b, c, d, g,	27.81 (23.70–33.90)	a, b, c, d, g,
f. Scrimmage	11.20 (4.54–17.86)	e, h	11.12 (5.81–16.39)	a, b, d,	30.20 (21.19–39.22)	a, c, d
g. Self-care	14.26 (9.79–18.81)	d, e, h	13.08 (9.96–16.24)	a, b, e, i	37.73 (31.23–44.17)	a, c, d, e, h
h. Sport-skill	17.58 (13.29–21.91)	a, c, d, e, f, g, i	10.73 (7.76–13.64)	a, b, c, d,	31.56 (25.52–37.68)	a, c, d, g
i. Strategy	12.58 (8.29–16.91)	d, e, h	8.48 (5.56–11.44)	b, c, d, g	30.62 (24.33–36.87)	a, c, d
	Low-energy stationary behavior	Differences^b^ (*p* < .05)	VPA	Differences^b^ (*p* < .05)	MVPA	Differences^b^ (*p* < .05)
Member Arrangement						
a. One v One	12.53 (4.46–20.54)	None	16.09 (9.63–22.57)	None	35.29 (24.72–45.88)	None
b. Small group	13.27 (7.12–19.28)	None	10.06 (5.20–15.00)	None	35.55 (27.37–43.83)	None
c. Whole group	15.52 (11.97–19.03)	None	12.47 (9.76–15.24)	None	34.53 (29.21–39.79)	None
	Low-energy stationary behavior	Differences^c^ (*p* < .05)	VPA	Differences^c^ (*p* < .05)	MVPA	Differences^c^ (*p* < .05)
Setting Demand						
a. Disadvantaged	18.76 (14.68–22.92)	b	10.30 (7.16–13.44)	b	29.07 (23.22–34.98)	b
b. Optimal	14.21 (10.67–17.73)	a	13.21 (10.65–15.75)	a	36.06 (30.81–41.39)	a

^**a**^Significance from mixed effects model (e.g.,‘a’ denotes difference from warm-up)

^b^Significance from mixed effects model (no significant differences found)

^c^Significance from mixed effects model (e.g.,‘a’ denotes difference from disadvantaged)

#### Task

Free-play segments had a significantly greater percentage of time in MVPA than fitness, scrimmage, sport-skill, and strategy segments. Gameplay segments had a significantly greater percentage of time in MVPA compared to fitness, sport-skill, and strategy segments. Warm-up segments had a significantly greater percentage of time in MVPA than fitness segments.

A significantly greater percentage of low-energy stationary behavior time was found in fitness segments than gameplay segments, sport-skill segments than in free-play, gameplay, and warm-up segments, and in strategy segments than in gameplay segments.

Free-play segments had significantly greater percentage of time in VPA than in sport-skill, and strategy segments. Gameplay segments had a significantly greater percentage of time in VPA than in sport-skill, strategy, and scrimmage segments. Fitness segments had a significantly greater percentage of time spent in VPA than sport-skill, strategy, and scrimmage segments.

#### Member arrangement

There were no significant differences for any physical activity intensity between member arrangement types.

#### Setting demand

Segments with an optimal setting demand had a significantly greater percentage of time in MVPA and VPA than disadvantaged demand segments, while disadvantaged segments had significantly higher percentage of time spent in low-energy stationary behavior than optimal segments. An additional exploratory analysis indicated that within segment task types, participant demand was associated with varying amounts of the percentage of time spent of MVPA and VPA. Optimal demand segments had higher MVPA and VPA within warm-up (MVPA, *t =* 3.25, *p =* 0.001; VPA, *t =* 3.25, *p =* 0.001) and skill (MVPA, *t =* 5.12, *p <* 0.001; VPA, *t =* 3.53, *p <* 0.001) than disadvantaged demand segments.

## Discussion

The present study segmented youth sport (YS) practice time by contextual characteristics, described the type and frequency of the segments, and examined the influence of time segments on MVPA. Our findings supported study hypotheses, that: (1) practices would have heterogeneous time segments defined by contextual characteristics, (2) member arrangement and setting demand of the time segment would influence MVPA, and (3) time segments that demanded participation (i.e., optimal demand) would result in greater MVPA compared to segments that fostered exclusion (i.e., disadvantaged demand).

Few other studies [9, 10, 33] have examined contextual variables during youth sport practice, although comparisons to the present study should be made cautiously. The direct observation systems used by Guagliano and colleagues [10] and Cohen and colleagues [9] both used momentary time-sampling techniques and sequentially followed a set of focal children to determine the average percentage of time spent in various activity intensities and various contexts across a whole practice. In contrast, the present system segmented the practice based on naturally occurring changes in context, rather than pre-determined time intervals, and followed a team, rather than an individual. In addition, some coding scheme variables had operational definitions that differed between the coding systems. For example, the current coding system and OSRAC-YS include a warm-up code, whereas SOFIT does not include a code for warm-up, but classifies any warm-up activities as fitness [29]. Further, in order to distinguish between the use of sport-related games and non-sport-related games (e.g., tag) in a YS practice, our system included a code for ‘scrimmage’, which would be classified as ‘gameplay’ using SOFIT [10] and OSRAC-YS [9].

Contrary to the present study, Cohen and colleagues [9] found that drills and fitness practice contexts (task in the present study) had the highest percentage of intervals spent in MVPA. This difference is likely attributable to differing observation methods described above. Cohen and colleagues also found small group and individual social contexts (participant arrangement in the present study) had the highest percentage of intervals spent in MVPA, whereas the present study found no difference in percentage of time spent in MVPA between participant arrangement types. This may be explained by the low variability of participant arrangements witnessed in the observed sport practices in the present study; 93% of segments observed were whole group.

**Fig. 2:**
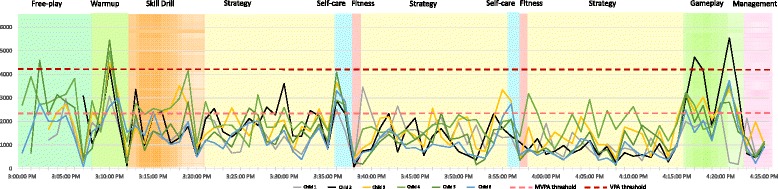
Physical activity and time segments of one team across one practice

To our knowledge, this is the first study to describe PA of segmented YS flag football practice. As depicted in Fig. 2, during a youth sport practice, there are peaks and valleys of activity that occur thereby creating a heterogeneous pattern of children’s PA. Averaging PA across an entire youth sport practice loses this pattern of variability, thus periods of time that are spent highly active or inactive cannot be distinguished from the whole practice. Identifying periods of time when activity is low during youth sport can help highlight key opportunities to intervene to increase PA during youth sport. Furthermore, by segmenting practice time based on naturally occurring changes in context, we were able to examine the ecological processes that contribute to driving low activity levels.

Our second hypothesis, that segments fostering participation would yield more MVPA than segments that fostered exclusion, was supported. Across all segment task and arrangement types, segments that fostered participation rather than exclusion resulted in higher levels of MVPA, VPA, and lower levels of low-energy stationary behavior time. Barker and Gump [20] first posited that ecological systems that were overloaded encouraged less participation from the whole group. Since then, classroom ecology has built on that premise, particularly in physical education [34]. The principle of setting demand as an ecological process has been applied in interventions across youth settings such as youth sport [35], physical education [36–38], and after-school programs [39] by training leaders to implement strategies such as using small groups, eliminating lines, and modifying elimination games in order to increase the amount of children engaged in physical activity at one time. In the youth sport setting, Guagliano and colleagues [35] demonstrated that a short-term coaching intervention focused on decreasing management time, eliminating lines, and modifying games and drills to offer more opportunities to be physically active was successful at increasing the percentage of time girls spent in MVPA during a basketball camp, compared to a standard-care control group. The present study offers additional support that activities fostering participation are associated with higher levels of MVPA. In the current study, segments that fostered participation had higher levels of MVPA and VPA than those that fostered exclusion; within the task type of warm-up and sport-skill, optimal demand segments had greater MVPA and VPA than disadvantaged segments of the same task type.

Youth sport contributed approximately 20 min of MVPA toward meeting current physical activity guidelines for children of 60 min of MVPA per day. Across all teams and segments, approximately 34% of practice time was spent in MVPA. Though there currently is no recommendation for the percentage of time children should spend in MVPA during youth sport, other youth activity settings, such as physical education, have been recommended to spend at least 50% of time in MVPA [40]. The present study indicates that segments with the highest percentage of time in MVPA were warm-up, gameplay, and free-play, respectively, all of which were above 50% of time. Of the 28 observed practices, however, there were only 16 occurrences of warm-up, and were even fewer segments of gameplay (*n* = 9), fitness (*n* = 8), and free-play (*n* = 7). In comparison, management and sport-skill segments had the highest percentage of time spent in low-energy stationary behavior and occurred 37 and 49 times, respectively. This suggests that inserting segments of task types shown to have a high percentage of time in MVPA (e.g., dynamic warm-up, playground games) into a youth sport practice routine and decreasing the frequency of segments with a high percentage of low-energy stationary behavior time could increase the percentage of time children spend in MVPA during practice. In addition, dynamic warm-ups have been recommended for injury prevention [41, 42] though less than two-thirds of practices in the present study included a warm-up segment. As youth sport has a large global reach and the potential to make a public health impact, future research should attempt to determine which practice components are most conducive to physical activity, without compromising other goals of a youth sport session (e.g., skill development) and how to train coaches to implement these components into their practice routines.

The present study is not without limitations. Our convenience sample was limited to 14 teams, included only boys, and we observed just two practices per team which creates the potential for selection bias. Additionally, although accelerometers have been shown to be a valid and reliable measure of youth PA, multiple methodological considerations for measurement of MVPA and VPA still lack scientific consensus. In contrast, the study boasts a number of strengths. This study presents a novel approach to combine objectively measured physical activity with direct observation of contextual variables in sport practice to characterize the pattern of PA across a youth sport practice and determine the influence of ecological processes on PA in the youth sport setting. The study had a high consent rate (91%) across all teams, accelerometer wear time and practice beginning and end times were rigorously defined, and the coding system had high inter-rater reliability.

## Conclusion

In conclusion, the current study suggests that PA during youth sport practice is highly variable across time and is driven by changes in context. Changes in task and setting demand were associated with changes in percentage of time spent in MVPA. Segments within a practice that foster participation are likely to drive practices to have a higher percentage of time spent in MVPA and VPA than those practices that foster exclusion. Further research is needed to identify the combination of types of segments that will optimize physical activity without compromising the other goals of youth sport, such as skill development. As youth sport reaches a large number of children world-wide, increasing the percentage of time spent in MVPA during setting time has the potential to create a public health impact.

## Abbreviations

%time: Percentage of time
CPM: Counts per minute
LPA: Light physical activity
MVPA: Moderate-to-vigorous physical activity
PA: Physical activity
VPA: Vigorous physical activity
YS: Youth sport
